# Origin of sample size effect: Stochastic dislocation formation in crystalline metals at small scales

**DOI:** 10.1038/srep39242

**Published:** 2016-12-15

**Authors:** Guan-Rong Huang, J. C. Huang, W. Y. Tsai

**Affiliations:** 1 Department of Materials and Optoelectronic Science, National Sun Yat-Sen University, Kaohsiung 804, Taiwan, ROC; 2 Physics Division, National Center for Theoretical Sciences, Hsinchu 30013, Taiwan, ROC

## Abstract

In crystalline metals at small scales, the dislocation density will be increased by stochastic events of dislocation network, leading to a universal power law for various material structures. In this work, we develop a model obeyed by a probability distribution of dislocation density to describe the dislocation formation in terms of a chain reaction. The leading order terms of steady-state of probability distribution gives physical and quantitative insight to the scaling exponent *n* values in the power law of sample size effect. This approach is found to be consistent with experimental *n* values in a wide range.

Experiments have shown that crystalline metallic materials can be strengthened by sample size reduction^1^,^2^, and this is so-called sample size effect governed by the empirical power law of scaling type, *s* = *d*^−*n*^, where *s* is the scaling stress, *d* is the characteristic length scale of the sample, and *n* is the scaling exponent. With the progress in experiments at small scales, researchers can make a fine measurement on the mechanical properties of metals. For the past decades, researchers have found that the range of *n* is about 0.6 to 0.7 for face-centered cubic (FCC) metals while 0.3 to 0.8 for body-centered cubic (BCC) metals^3^,^4^,^5^,^6^,^7^,^8^,^9^,^10^,^11^. Recent studies also suggest that *n* lies within 0.3 to 1.0^12^. The general trend held for micro- to mini-scale seems to have nothing to do with crystal structure and sample geometry^2^,^13^,^14^,^15^,^16^, which represents there should be a universal mechanism underlying in different metals.

The size effect of metals can be explained by using statistical techniques^17^,^18^,^19^, namely, the stress or strain occurrs in stochastic events with some underlying probability distributions. The relation between stress and dislocation density is the double power law form during the process of dislocation source activation and/or dislocation forest hardening for different sample sizes and initial dislocation density^20^,^21^. Therefore, the mechanism of size effect is dependent on the dislocation density. Before applying stress, crystalline metals with a high degree of perfection at small scales possess a relatively lower order of dislocation density (about 10^4^ *cm*^−2^). Over extensive studies, it has been widely acknowledged that slip events occurred in crystals are conducted with the aid of dislocations. The plastic deformation in crystals corresponds to a large amount of dislocation formations, dislocation interactions and recoveries, which are generally regarded to be initiated from the Frank-Read or similar sources.

Experimental evidence shows that the Frank-Read or similar sources can act as the dislocation nucleation sites. For the sake of simplicity, homogeneous and heterogeneous dislocation nucleation are both merged as parts of dislocation nucleation, and they may generate a dislocation or recover to their original state. Additionally, experiments show that dislocation nucleation is driven by stress and diffusion-controlled process^12^,^22^,^23^. After dislocation nucleation, mobile dislocations can move and increase their length to accomplish the shape changes in metallic crystals, and hence the dislocation density will be increased. This increases the possibility of dislocation interactions leading to dislocation recovery. Thus, Fig. 1 exhibits the universal mechanism for dislocation formation in different metals to describe the dynamics of dislocation density and to relate the physical meaning of scaling exponent *n* in sample size effect to experimental observations. In Fig. 1, *k*_1_ is the dislocation nucleation rate of nucleation site into dislocation nucleation driven by stress, *k*_2_ is the rate of dislocation formation after nucleation into dislocation multiplication, where mobile dislocations can glide and increase their length, *γ*_1_ is the recovery rate of dislocation nucleation, *γ*_2_ is the recovery rate of dislocation, and *D* is the dislocation nucleation rate of nucleation site into dislocation nucleation driven by diffusion process. Then it will be considered in later section that the overall generation of dislocation is characterized by two parameters: the effective mean number of dislocation nucleation during the life time of dislocation, *α* = (*k*_1_ + *D*)/*γ*_2_, and the mean number of dislocation multiplication during the life time of dislocation nucleation, *β* = *k*_2_/*γ*_1_. The life time of nucleation is assumed to be short compared to that of dislocation, where the plastic events can be counted as the uncorrelated random events. In a word, dislocations are created by the plastic deformation plus thermally-activated diffusion.

Since these events increasing the dislocation density are all stochastic processes, the stochastic differential equation of PD in terms of dislocation density and time can describe the dynamics of dislocation density. With the known dynamics of PD, the dynamics of stress can be established through the relation between dislocation density and stress. With the known PD in Eq. (7), the trajectory of plastic deformation can be counted as a sequence of steady-state dislocation density, which gives the *n* value via the algorithm in refs 17,18 and exponent *τ* value in ref. 19.

## Dislocation Stochastic Formation and Scaling Exponent

**Figure 1:**
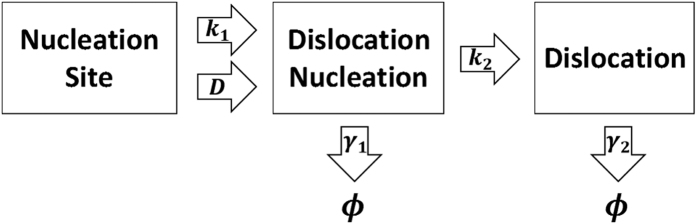
The mechanism of dislocation formation driven by the plastic events plus thermally-activated diffusion process on single crystal metals at small scales.

To investigate the dynamics of dislocation network, we can introduce a master equation, the equation of motion for PD. In ref. 24, the simple mechanism in Fig. 1 with a fluctuation term can be described by the stochastic differential equation in terms of dislocation density *ρ* and time *t*


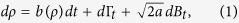


where *b*(*ρ*) is the deterministic term, Γ_*t*_ is the stochastic process driven by plastic deformation, and *B*_*t*_ is the fluctuation term. Γ_*t*_ is compound noise with impulsive Poisson distribution, and *B*_*t*_ is Gaussian noise and fluctuation around a global extrema. Both they are stochastic processes with independent increment. Equation (1) is size-dependent^25^,^26^, since the mechanism of dislocation source activation and dislocation forest hardening depends on the initial dislocation density and sample size. Common deformation tests are conducted at nearly constant temperature, the temperature effect on reaction rates can be neglected during tests. The corresponding master equation of Eq. (1) for the PD *P*(*ρ, t*) is expressed as


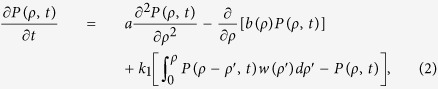


where the *a* value corresponding to *B*_*t*_ is the magnitude of fluctuation, the integration term corresponding to Γ_*t*_ can be counted as the reaction of dislocation formation induced by plastic deformation, and *w*(*ρ*′) is the average transition probability density from dislocation nucleation into dislocation in the material. In the integration term of Eq. (2), 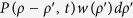 is the transition probability from *ρ* − *ρ*′ to *ρ*. Hence, it is reasonable to assume *w*(*ρ*′) as the exponential decay function, 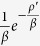. On the other hand, the annihilation rate of dislocation increases with increasing dislocation density due to the attraction among opposite signed dislocations and the reduction of overall dislocation strain energy^27^,^28^. Thus, the recovery term is set as *b*(*ρ*) = −*γ*_2_*ρ*. Therefore, Eq. (2) with such *b*(*ρ*) and *w*(*ρ*′) is re-written as


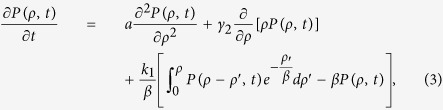


and the corresponding mean of dislocation density with time evolution is


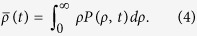


For the steady-state of Eq. (3), 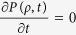, the solution solved in ref. 29 is expressed as the combination of the Kummer’s functions. In ref. 20, flow stress is connected to dislocation density in double power law form


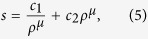


where *c*_1_ is inverse proportional to sample size, *c*_2_ is a constant, and 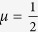. There exists a critical dislocation density as a function of sample size, *ρ*_*c*_ = *c*_1_/*c*_2_, where it is the transition between dislocation source activation and dislocation forest hardening^20^,^30^,^31^,^32^. Below *ρ*_*c*_ the mechanism of dislocation source activation will gradually become dominant with decreasing dislocation density. Likewise, above *ρ*_*c*_ the mechanism of dislocation forest hardening or dislocation multiplication will gradually become dominant with increasing dislocation density. For *ρ* away from *ρ*_*c*_, the relation of *s* and *ρ* is simple power law form, and thus 
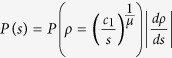
 for 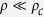 or 
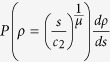
 for 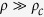. In general cases, the *a* value is much smaller than the *γ*_2_ value, and thus the *a* term of Eq. (2) can be neglected without loss of generality. Therefore, *P*(*ρ*) without the *a* term of Eq. (3) solved in ref. 33 is


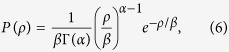


and the corresponding PD in terms of *s* is


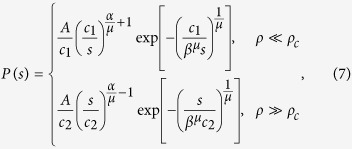


where *A* = [*μβ*^*α*^Γ(*α*)]^−1^. The second line in Eq. (7) is the form of generalized gamma distribution which has been used in statistical simulation for stress dynamics in refs 17,18. Finally, the dislocation density of steady-state *ρ*_*s*_ is


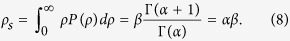


For common deformation tests, the strain rate is a small value in the 10^−4^ *s*^−1^ ~ 10^−5^ *s*^−1^ order. The strain rate is so slow that the deformation process can be counted as a series of steady state processes corresponding to different *β* values. Therefore, the deformation process can be characterized as a series of *β* values or *ρ*_*s*_ values like the sequence: {*β*_1_, *β*_2_, …} or 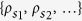, where the different *β* and *ρ*_*s*_ values correspond to different steady-state processes.

With the aid of refs 17, 18, 19,34, the scaling exponent *n* can be constructed through *P*(*s*)


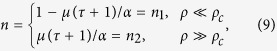


where *τ* is the exponent in the envelope distribution for the normalized strain in refs 19,35. The *α* values in the two lines of Eq. (9) are different since they correspond to different mechanisms, dislocation source activation and dislocation forest hardening. For the intermediate region, *ρ* < *ρ*_*c*_ or *ρ* > *ρ*_*c*_, the asymptotic relation of *s* and *ρ* can be obtained by binomial expansion


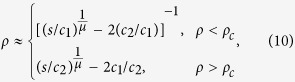


which gives the scaling exponent


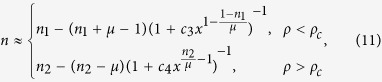


where *c*_3_ and *c*_4_ are both constants and *x* = 1/*d*.

## Discussion

For crystalline metals at small scales, we consider the master equation of PD as a function of dislocation density, where dislocations are produced by the deformation of the material plus diffusion processes. In the material, the generation and annihilation of dislocations are characterized by the constants of reaction rate: *k*_1−2_ and *γ*_1−2_. As time past, the mean of dislocation density governed by Eq. (4) will reach a critical value *ρ*_*s*_ in Eq. (8), where the plastic event occurs and the dislocation density of steady-state is characterized by the parameter, *αβ* = (*k*_1_ + *D*)*k*_2_/*γ*_1_*γ*_2_. It is considered as chemical equilibrium, where the ratio of forward reaction rate (*k*_1_ + *D*)*k*_2_ to backward reaction rate *γ*_1_*γ*_2_ is a constant. The double power law relation of *s* and *ρ* has been used to construct the PD in terms of stress *s*. The different stress and *β* values correspond to different steady-state dislocation density, and the deformation process can be counted as a sequence of dislocation density, 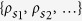.

**Figure 2:**
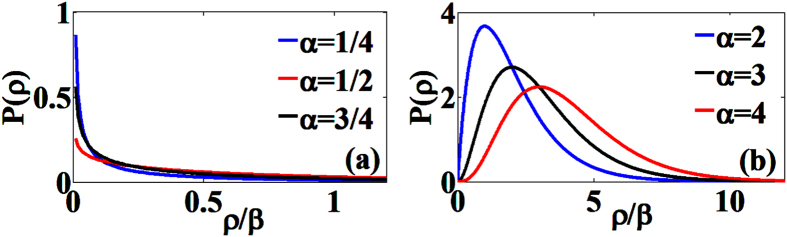
(**a**) The steady-state PD predicted by Eq. (6) for *α* < 1 with *β* = 10. (**b**) The steady-state PD predicted by Eq. (6) for *α* > 1 with *β* = 10 and the unit of y-axis is 10^−2^.

**Figure 3:**
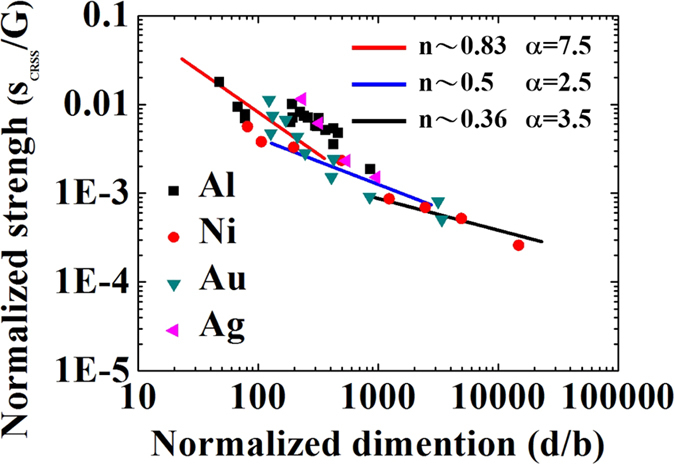
The comparison between experimental results and *n* values predicted inEq. (9), where 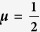 and 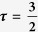 are used. The data points were extracted from experiments in refs 1,2,8, 9, 10,16,37, 38, 39 with different single crystal metals listed.

For many crystalline metals at small scales, the *τ* is 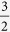, 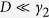, and 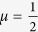 corresponding to 
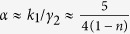
 or 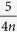 and 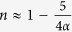 or 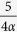, depending on the dislocation density and sample size. The *n* value is only sightly altered by diffusion nucleation. In this study, *α* can vary from 0 to ∞, depending on the relative weighting of *k*_1_ + *D* versus *γ*_2_. For example, if *α* = 0.25, 0.5, 1.5, 2.5, 3.5, and 4.5, *n* would equal −4, −1.5, 0.17, 0.5, 0.64, and 0.72 for 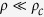 or 5, 2.5, 0.83, 0.5, 0.36, and 0.28 for 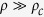. According to experiments, the general cases are approximately in the regime of 0.75 ≤ *n* ≤ 0.85 for 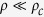 or 0.3 ≤ *n* ≤ 0.4 for 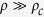, resulting in 5 ≤ *α* ≤ 8.3 for 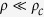 or 3.1 ≤ *α* ≤ 4.2 for 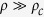. As stated before, *α* is defined as the effective mean number of dislocation nucleation, so the physical meaning of *α* can be related to the activation of dislocation sources or the average number of active slip systems. In the regime of initial dislocation density above *ρ*_*c*_, the mechanism is mainly dislocation forest hardening or dislocation multiplication, and thus only dislocations nucleated right on these planes of active slip systems will be increased in length drastically, while dislocations nucleated on other planes may die out through dislocation recovery or keep in relatively small length since these dislocations can not glide to increase their length. Thus, the *α* value should be proportional to the average number of active slip systems. For metal crystals, the number of active slip systems is from 2 to 5 depending on the crystal structure, FCC or BCC. Therefore, the *α* < 1 case would not be realistic, meaning that no slip system is well-activated. For a metal with more active slip systems, it has weaker size effect, and vice versa. Generally, BCC metals with less active slip systems would have higher *n* (lower *α*) value than FCC metals with more active slip systems would have. In ref. 36, the *n* value is related to initial dislocation density, samples with more dislocation density would have lower *n* value. Here, it means that a metal with more initial dislocation density would have higher *α* value. Since initial dislocations have occupied more sites which may be randomly distributed in space, some of these sites are on the planes of active slip systems. Thus, there exists more effective sites for dislocation nucleation (higher *α*), and vice versa. In the regime of initial dislocation density below *ρ*_*c*_, the mechanism is mainly dislocation source activation which leads to dislocation source strengthening, and thus *n* will increase with increasing *α*. Low *α* values would not be taken as real cases, since the dislocation formation mainly comes from dislocation nucleation without dislocation multiplication. For the initial dislocation density in intermediate regime, 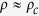, there is a competition among source strengthening, forest hardening, and exhaustion hardening. It is hard to determine the accurate form of *n* due to the complex transitions among these mechanisms. Equation (11) gives the asymptotic behaviour of *n* in first order approximation, and it can be noted that *n* and *ρ*_*c*_ will both increase with decreasing *d*. It is the fact that dislocation source strengthening will gradually become dominant with decreasing *d*, and thus the *n* value will be increased. The mechanism governed by Eq. (3) is random and uncorrelated events, and it leads to the PD in terms of *s* for 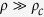, which is the same form of PD in refs 17,18, where their PD are generalized gamma distributions. For both 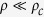 and 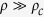 regimes, the leading order term of PDs are proportional to 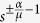. As shown in Fig. 2, the PD is characterized by the *α* value, and thus dislocation generation is strongly affected by the dislocation source activation and the average number of active slip systems. For *α* < 1 cases corresponding to *n* < −0.25 for 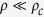 or *n* > 1.25 for 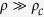, the dislocation annihilation rate is larger than dislocation nucleation rate, and thus the maximum of distribution is located at *ρ* = 0. For *α* > 1 cases corresponding to *n* > −0.25 for 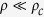 or *n* < 1.25 for 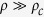, the maximum of distribution is located at *ρ* = *β*(*α* − 1), where *α* is increased by dislocation source activation, or the active slip systems for dislocations are initiated with a high effective number of dislocation nucleations. Figure 3 shows the double-logarithm plot for normalized strength and normalized sample size extracted from refs 1,2,8, 9, 10,16,37, 38, 39, where the *n* and *α* have different values across different regimes: dislocation source activation (*n* ~ 0.83 and *α* ~ 7.5), transition from dislocation source activation to dislocation forest hardening (*n* = 0.5 and *α* ~ 2.5), and dislocation forest hardening (*n* = 0.36 and *α* ~ 3.5). For the present of small *a* term, the maximum point and general shape of *P*(*ρ*) does not change, and it only changes the *P*(*ρ*) value at *ρ* = 0 to a finite value^29^. Thus, the general form of Eq. (6) will not change. Our analytic results straightforwardly provide a microscopic theory and mechanism in understanding the physical insight of *α* and the universal power law in sample size effect.

## Additional Information

**How to cite this article**: Huang, G.-R. *et al*. Origin of sample size effect: Stochastic dislocation formation in crystalline metals at small scales. *Sci. Rep.* **6**, 39242; doi: 10.1038/srep39242 (2016).

**Publisher’s note:** Springer Nature remains neutral with regard to jurisdictional claims in published maps and institutional affiliations.
